# Bridgman-Grown (Cd,Mn)Te and (Cd,Mn)(Te,Se): A Comparison of Suitability for X and Gamma Detectors

**DOI:** 10.3390/s24020345

**Published:** 2024-01-06

**Authors:** Aneta Masłowska, Dominika M. Kochanowska, Adrian Sulich, Jaroslaw Z. Domagala, Marcin Dopierała, Michał Kochański, Michał Szot, Witold Chromiński, Andrzej Mycielski

**Affiliations:** 1 Institute of Physics, Polish Academy of Sciences, Aleja Lotników 32/46, 02-668 Warsaw, Poland; dmkoch@ifpan.edu.pl (D.M.K.); sulich@ifpan.edu.pl (A.S.); domag@ifpan.edu.pl (J.Z.D.); mardop@ifpan.edu.pl (M.D.); mkochanski@ifpan.edu.pl (M.K.); szot@ifpan.edu.pl (M.S.); 2 International Research Centre MagTop, Institute of Physics, Polish Academy of Sciences, Aleja Lotników 32/46, 02-668 Warsaw, Poland; 3 Faculty of Materials Science and Engineering, Warsaw University of Technology, Wołoska 141, 02-507 Warsaw, Poland; witold.chrominski@pw.edu.pl; 4 Puremat Technologies Sp. z o.o., Aleja Lotników 32/46, 02-668 Warsaw, Poland

**Keywords:** annealing, crystal growth, photoluminescence, X-ray and gamma-ray detectors, X-ray diffraction, XRD

## Abstract

This study explores the suitability of (Cd,Mn)Te and (Cd,Mn)(Te,Se) as room-temperature X-ray and gamma-ray detector materials, grown using the Bridgman method. The investigation compares their crystal structure, mechanical and optical properties, and radiation detection capabilities. Both crystals can yield large-area single crystal samples measuring approximately 30 × 30 mm^2^. In low-temperature photoluminescence analysis, both materials showed defect states, and annealing in cadmium vapors effectively eliminated donor–acceptor pair luminescence in (Cd,Mn)Te but not in (Cd,Mn)(Te,Se). Moreover, harder (Cd,Mn)(Te,Se) exhibited a higher etch pit density compared to softer (Cd,Mn)Te. X-ray diffraction examination revealed uniform lattice constant distribution in both compounds, with variations at a part per million level. (Cd,Mn)Te crystals demonstrated excellent single crystal properties with narrower omega scan widths, while (Cd,Mn)(Te,Se) exhibited a high contribution of block-like structures with significantly larger misorientation angles. Spectroscopic evaluations revealed better performance of a pixelated (Cd,Mn)Te detector, in comparison to (Cd,Mn)(Te,Se), achieving a mean full width at half maximum of 14% for the 122 keV gamma peak of Co-57. The reduced performance of the (Cd,Mn)(Te,Se) detector may be attributed to deep trap-related luminescence or block-like structures with larger misorientation angles. In conclusion, Bridgman-grown (Cd,Mn)Te emerges as a more promising material for X-ray and gamma-ray detectors when compared to (Cd,Mn)(Te,Se).

## 1. Introduction

CdTe-based compounds such as (Cd,Zn)Te [1,2,3,4], (Cd,Zn)(Te,Se) [5,6,7,8,9,10,11,12,13,14,15,16,17,18], (Cd,Mn)Te [19,20,21,22,23,24], (Cd,Mn)(Te,Se) [25,26], (Cd,Mg)Te [27,28,29,30,31,32], and Cd(Te,Se) [33,34,35,36,37,38,39] are currently being tested as materials for room-temperature X- and gamma-ray detectors. In these nuclear detectors, it is crucial to maximize the flow of radiation-induced charge carriers through the detector volume to the respective electrodes. Therefore, it is highly desirable for the material to have minimal defect concentrations to ensure optimal detector performance. In practice, this translates to the requirement of high resistivity (ρ~10^9^–10^10^ Ω cm) and a high mobility-lifetime product (μτ~10^−3^ cm^2^V^−1^) for the crystals [40].

In our laboratory, we regularly conduct investigations on high-resistivity (Cd,Mn)Te, which is the material we proposed for X-ray and gamma detectors [41]. This material has demonstrated considerable potential in the aforementioned application [42]. Furthermore, for crystal growth, we employ the Bridgman method [43,44], enabling the relatively fast production of large (≥1.5 inch) crystals with a crystal growth rate of several millimeters per hour, as compared to techniques such as the traveling heater method (THM), where the crystal growth rate is 3–5 mm per day [45,46]. Our motivation to study compounds alloyed with selenium, specifically (Cd,Mn)(Te,Se), stemmed from the literature findings [10]. In the field of X-ray and gamma-ray detectors, there is currently a heated debate regarding the use of selenium-alloyed CdTe crystals [5,6,7,8,9,10,11,12,13,14,15,16,17,18,25,26]. In this article, we present new data and express our position on this matter.

According to previous research on (Cd,Zn)Te and (Cd,Zn)(Te,Se) [8,47], the presence of selenium plays a crucial role in inhibiting the development of sub-grain boundary networks and increases the hardness of the quaternary material (Cd,Zn)(Te,Se). Additionally, selenium significantly reduces the concentration of tellurium inclusions [46]. Subsequently, it can be inferred from UV–Vis absorbance data that changes in the bandgap values along the growth direction (reduced length of 0.1 to 0.9 of the ingot) in Cd_0.9_Zn_0.1_Te crystals amount to 31 meV, whereas in Cd_0.9_Zn_0.1_Te_0.98_Se_0.02_, they are only 21 meV [48]. This indicates that in (Cd,Zn)(Te,Se) crystals, a more uniform difference of bandgap values along the growth direction is achieved due to reduced segregation effects. A homogeneous distribution of the energy bandgap in crystals is important because it influences the distribution of other properties, such as the resistivity and absorption edge [39].

In this work, instead of the more commonly investigated (Cd,Zn)Te, we utilize (Cd,Mn)Te due to several advantages of Mn-alloyed CdTe over Zn-alloying. The first benefit is related to segregation, which is a shift in compositions of liquid and solid phases in thermodynamic equilibrium that occurs during the crystallization process. It is defined by a segregation coefficient, which is a ratio of the concentration of an element in a solid to the concentration of an element in a liquid [49]. The segregation coefficient of Mn in CdTe is close to unity, specifically 0.95 [50], whereas the segregation coefficient of Zn in CdTe is 1.3 [51]. This results in a more homogeneous distribution of Mn in CdTe, whereas in (Cd,Zn)Te, there is a higher concentration of Zn at the first-to-freeze part of the ingot. However, there are reports that the addition of Se to (Cd,Zn)Te reduces Zn segregation in the ingot, and a homogeneous chemical composition can be achieved in the axial and radial directions in 90% of the volume of (Cd,Zn)(Te,Se) crystals obtained using the THM method [46] and horizontal Bridgman technique [52]. Secondly, for a detector to operate at room temperature, the semiconductor material should have an appropriate energy bandgap, ranging from 1.5 to 2.2 eV [40]. Achieving the desired energy bandgap involves adding a larger quantity of Zn to CdTe in comparison to introducing Mn to CdTe. This is because the energy bandgap of (Cd,Zn)Te undergoes a slower transformation with the addition of Zn, changing at a rate of 6.7 meV per atomic percent of Zn [53]. Conversely, introducing Mn to CdTe alters the energy bandgap by 13 meV for every atomic percent of Mn [54]. Furthermore, the addition of Se to CdTe reduces the energy bandgap in crystals typically chosen for X-ray and gamma-ray detector applications, specifically those containing less than or equal to 2% Se. In CdTe_1−x_Se_x_ crystals, the energy gap decreases as x increases, at a rate of approximately 4 meV per atomic percent of Se for x ≤ 0.1. This rate decreases for 0.1 ≤ x ≤ 0.4, reaches its minimum at x ≈ 0.4, and then begins to increase as x continues to rise [55]. This consideration is significant. Restricting the quantity of the third or fourth alloying component helps limit the formation of new defects within the crystal structure. Thirdly, it has been experimentally observed that larger grains can be obtained in (Cd,Mn)Te than in (Cd,Zn)Te, enabling the production of larger volume detector plates from such a crystal.

In this study, we investigate two compounds grown by the Bridgman method: (Cd,Mn)Te and (Cd,Mn)(Te,Se), and we compare their suitability for X-ray and gamma-ray detectors. The research involved examining the hardness and employing the etch pit density technique to determine whether harder materials display fewer detrimental sub-grain boundaries and their networks. We conducted microstructure imaging using infrared microscopy. Subsequently, we performed a detailed characterization of the crystalline structure of selected large-surface-area single-crystal samples, such as 30 × 30 mm^2^. We drew lattice constant and omega scan maps for these compounds. The photoluminescence of the as-grown samples has been investigated, and by comparing the results with samples annealed in Cd and Se vapors, we discuss the presence of defects in the as-grown crystals. Finally, we present the energy spectrum from a Co-57 source recorded using our selected and optimized crystal.

## 2. Materials and Methods

Crystals of Cd_1−x_Mn_x_Te and Cd_1−x_Mn_x_Te_1−y_Se_y_ were grown using the low-pressure Bridgman method [56], with x set at either 5% or 7%, and y at 2%. The growth process employed high-purity materials, specifically 7N Cd, 7N Te, 6N Mn, and 6N Se. These crystals had diameters of either 2 or 3 in. Hardness investigations and infrared microscopy observations were carried out on a Cd_0.95_Mn_0.05_Te_0.98_Se_0.02_ crystal as the primary sample, while Cd_0.93_Mn_0.07_Te_0.98_Se_0.02_ crystals were utilized in other measurements. In the context of (Cd,Mn)Te crystals, those with a 5% Mn composition were consistently examined. The crystal growth process was performed under Te-rich conditions, involving the addition of 30 mg to 100 mg of extra Te per 100 g of material. For compensation, a co-doping of indium and vanadium was used. The concentration of indium was 1 × 10^17^ cm^−3^ (for Cd_0.95_Mn_0.05_Te), or 5 × 10^16^ cm^−3^ (for Cd_0.95_Mn_0.05_Te_0.98_Se_0.02_), or 1 × 10^14^ cm^−3^ (for Cd_0.93_Mn_0.07_Te_0.98_Se_0.02_), while that of vanadium was 1 × 10^13^ cm^−3^ for all crystals. To visualize the grain boundaries in the crystals, they were etched using the Durose solution [57]. Next, the samples were mechano-chemically polished using a 2% bromine solution in methanol and ethylene glycol.

Annealing processes were carried out for 168 h in vacuum-sealed quartz ampoules in Cd vapors at a temperature of 800 °C or in Se vapors at 350 °C.

Mechanical properties were tested by measuring the microhardness of the polished samples on the cadmium side, i.e., the (111)A plane, using a Vickers indenter with a load of 50 g for 15 s. For the purpose of comparison, one CdTe crystal, one Cd(Te,Se) crystal with a 5% Se composition, and two (Cd,Zn)Te crystals with 5% and 12% Zn, respectively, were also utilized in these studies.

The etch pit density was examined using the E-Ag1 Inoue solution, which consists of 0.5 parts AgNO_3_ and 10 parts solution E. Solution E is composed of 5 parts HNO_3_, 10 parts H_2_O, and 2 parts K_2_Cr_2_O_7_ [58]. The etching time was equal to 90 s. The (111)A side was observed. For microscopic observations, an Olympus BX51 microscope equipped with an Olympus XC10 CCD camera was utilized, either in reflection or infrared transmission mode, depending on the purpose.

In the diffraction studies, a high-resolution Philips X’Pert MRD diffractometer (Philips Analytical X-Ray B.V., Almelo, The Netherlands) was employed, featuring a monochromatized source of CuK_α1_ radiation (λ = 1.5406 Å) and further equipped with a homemade mask and slit. This homemade mask was responsible for reducing the dimensions of the X-ray beam to 0.5 × 1.0 mm^2^, enabling the collection of diffraction data from the samples point by point and line by line. X-ray scans were performed on sample areas measuring 18 × 20 mm^2^. Bragg angle measurements were carried out, with a focus on the 333 reflex, to generate maps of lattice constant variations within the samples. Omega curve measurements, depicting the intensity of the diffracted beam on the crystal as a function of the omega angle, ω, which is the angle between incident X-ray beam and the surface of the sample, were conducted to create delta omega maps and full width at half maximum (FWHM) maps. Both Bragg angle and omega curve measurements were carried out with a step size of 1 mm for X and 2 mm for Y.

Omega curve measurements were conducted in either double-axis mode (DA) or triple-axis mode (TA). In the TA mode, an analyzer was utilized to enhance the resolution, reducing it from 18 arcsec (DA) to 8 arcsec (TA), as determined by the measurement of a Si(111) reference sample. In the DA mode, the detector was fully opened. The omega angle measured with the analyzer is referred to as ω_TA_, while without the analyzer, it is denoted as ω_DA_.

The omega angle was determined at the peak of the curve or, in cases of multiple peaks, at the extreme peaks. Delta omega, Δω, was then calculated by subtracting these values from each other. In instances where only one peak was present on the curve, it was assumed that delta omega equaled zero. These data were utilized to generate delta omega maps.

The photoluminescence (PL) studies were carried out on the cleaved samples and the excitation energy was equal to 2.33 eV. The PL spectra were obtained at 5 K in a continuous flow cryostat with a photomultiplier.

The spectroscopic response at room temperature of the pixelated detectors was checked using a Co-57 source and a Eurorad spectroscopic pixel mapping machine (Eckbolsheim, France). For this purpose, electrical contacts, made of a gold-palladium alloy in an 80:20 ratio, were deposited using the Quorum Sputter Coater Q150T (Quorum Technologies, Laughton, United Kingdom). Pixels on the anode, which crystallographically represents the cadmium side, were produced using the photolithography method. The cathode remained planar. The sample surfaces were passivated using an aqueous solution of 10% weight NH_4_F and 10% weight H_2_O_2_ for 15 min [13,59]. The detector was illuminated from the cathode side and was maintained at a bias voltage of −400 V. The shaping time was set to 1 μs. The thickness of the samples used in these measurements ranged from 3 to 5 mm.

## 3. Results

**Figure 1:**
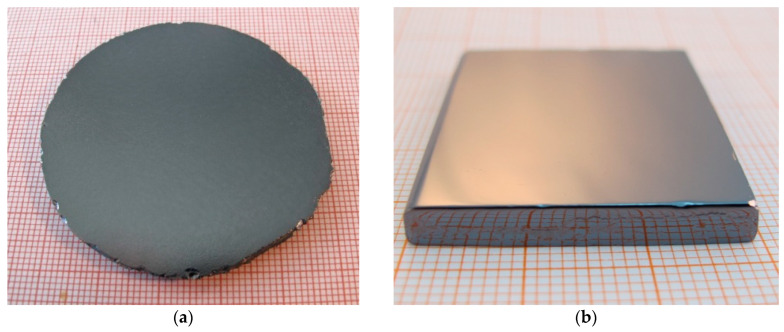
The image of the single-crystal (Cd,Mn)Te samples. (**a**) Crystal plate cut from the 2 in. ingot and etched with Durose solution. No grain boundaries or twins are visible. (**b**) (111)-oriented polished monocrystalline (Cd,Mn)Te plate of a specified shape for a detector. It was cut from a 3 in. ingot.

In our laboratory, by properly doping with In, V, or both In and V, we can obtain (Cd,Mn)Te and (Cd,Mn)(Te,Se) crystals with average resistivity on the order of 10^9^ Ω cm and a mobility-lifetime product on the order of 10^−3^ cm^2^V^−1^ [42]. We are also capable of producing large single-crystal plates, as depicted in Figure 1. Figure 1a shows a sliced (Cd,Mn)Te plate, which was cut perpendicular to the growth axis from a 2 in. ingot, ground, and then etched with Durose solution to reveal grain boundaries. It is evident that the plate is monocrystalline. On the other hand, Figure 1b shows a (111)-oriented polished plate that has been prepared for detector fabrication, i.e., for the application of electrical contacts. This plate is also monocrystalline and has large dimensions of approximately 30 × 30 mm^2^. Visual examination of the obtained crystals yielded satisfactory results. These results provide an excellent basis for further research, as the field of room-temperature X-ray and gamma-ray semiconductor detectors demands high-resistivity (>10^9^ Ω cm), defect-free, monocrystalline, large (more than 5–8 cm^2^), and sufficiently thick (>3 mm) plates for effective operation, i.e., to ensure effective interactions between high-energy radiation and the detector material. In Section 3.2, we will present more advanced studies on the crystal structure.

### 3.1. Hardness

**Figure 2:**
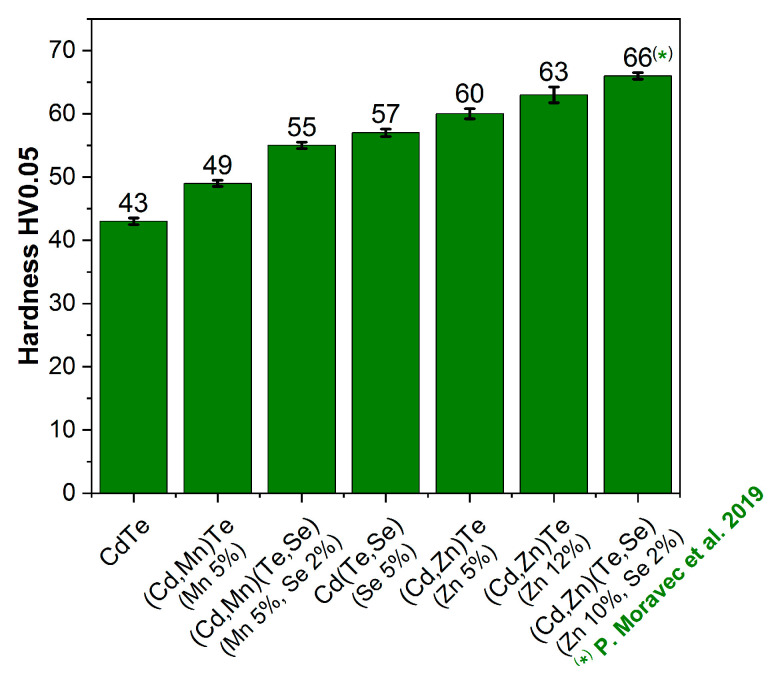
Hardness of the selected CdTe-based compounds measured on the (111)A cadmium side. The hardness value of the (Cd,Zn)(Te,Se) crystal was taken from reference [11].

The influence of Mn and Se additives on the hardness of the formed CdTe-based compounds was examined. As shown in Figure 2, a five percent addition of manganese or selenium to CdTe increases the hardness of the compound, with the influence of selenium being stronger. The hardness of CdTe alloyed with manganese (5%) and selenium (2%), i.e., (Cd,Mn)(Te,Se) compound, falls between the hardness of (Cd,Mn)Te (Mn 5%) and Cd(Te,Se) (Se 5%). For comparison, we also investigated two crystals of (Cd,Zn)Te with different Zn contents, namely 5% and 12%. The crystals with Zn exhibited the highest hardness among all the samples examined, and a higher Zn content resulted in increased hardness, which is consistent with the literature data [60]. In the comparison, we included the hardness result for CdTe crystal both alloyed with Zn and Se, measured by other authors [11]. The addition of 2% Se to (Cd,Zn)Te further hardened the material. The result maintains the same trend. Despite the greater hardness of (Cd,Zn)Te crystals, the focus of this study lies on (Cd,Mn)Te crystals because, as mentioned in Section 1, it is easier to attain larger grains in the latter.

The addition of some extra elements to the CdTe matrix, like Mn, Se, or Zn, alters its structure. These changes can make it more challenging for atoms and dislocations to move within the lattice, resulting in material hardening. Furthermore, by introducing an additional element to the CdTe matrix, the bond lengths, like Cd-Te, Mn-Te, Zn-Te, Cd-Se, are changed, leading to the formation of strong and stable atomic connections [61]. These bonds can hinder atom movement and impede material deformation.

### 3.2. Crystal Quality

#### 3.2.1. Etch Pit Density

A test was conducted to examine whether higher hardness values translate into a smaller population of sub-grain boundaries, which are often encountered in CdTe-based compounds produced using the Bridgman method and pose a significant issue in detector performance. Typically, sub-grain boundaries are investigated using the White Beam X-ray Diffraction Tomography method [8,62,63,64,65,66]. We employed an etching method to reveal etch pits using the Inoue solution, as the Nakagawa solution [67] yielded no results on our samples. Sub-grain boundaries, which have a small misorientation angle, are formed by dislocation clusters. Sub-grain sizes are on the order of hundreds of micrometers [63]. Hence, if we observe any clusters of etch pits, which form on the crystal surface at the location where dislocations initiate, it could suggest the presence of sub-grain boundaries in the investigated crystal. The formation of etch pits related to dislocations occurs due to the interplay between the stress field caused by dislocations and the surface energy [68].

**Figure 3:**
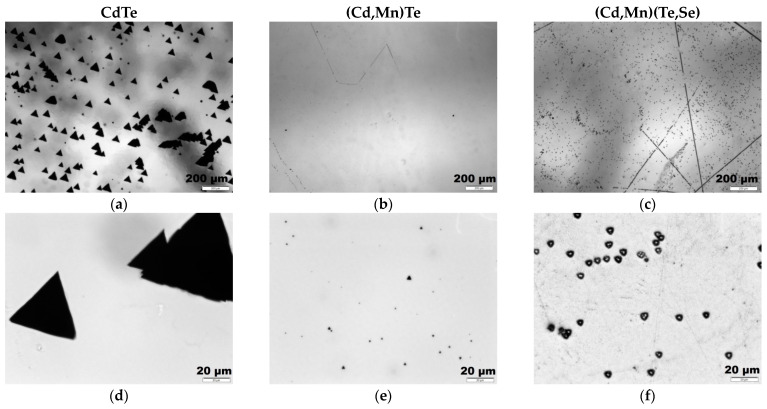
Etch pit density (EPD) images of CdTe—(**a**,**d**), Cd_0.95_Mn_0.05_Te—(**b**,**e**), and Cd_0.93_Mn_0.07_Te_0.98_Se_0.02_—(**c**,**f**). The tests were performed using the Inoue etchant on the ~(111)A plane. Subfigures (**d**–**f**) are magnified 10 times compared to subfigures (**a**–**c**), respectively.

Figure 3 illustrates microscopic images of the ~(111)A surface of three investigated by us compounds, which were etched with the Inoue solution to visualize etch pits. Figure 3a,d depict the surface of a CdTe reference sample at different magnifications. Figure 3b,e show (Cd,Mn)Te; Figure 3c,f represent (Cd,Mn)(Te,Se). The straight lines in Figure 3c represent scratches on the sample surface. The CdTe and (Cd,Mn)(Te,Se) samples exhibit a high density of etch pits, on the order of 10^5^ cm^−2^. However, in the CdTe sample, the average size of etch pits is significantly larger compared to (Cd,Mn)(Te,Se), measuring approximately 40 μm and 5 μm, respectively. The density of the etch pits is the lowest in the (Cd,Mn)Te sample, namely 10^4^ cm^−2^, and their size ranges between 3 and 5 μm. The larger size of the etch pits in CdTe than in (Cd,Mn)Te and (Cd,Mn)(Te,Se) may be attributed to the larger stress fields generated by dislocations in that region. Observations of the etch pits revealed their uniform distribution, without the formation of clusters resembling small-angle boundaries. This suggests the absence of sub-grain boundaries in each of the investigated compounds, although this cannot be conclusively determined by this method. However, it is certain that the (Cd,Mn)Te sample exhibited the smallest dislocation density on its surface and demonstrated the best quality among the samples examined.

#### 3.2.2. Lattice Constant

**Figure 4:**
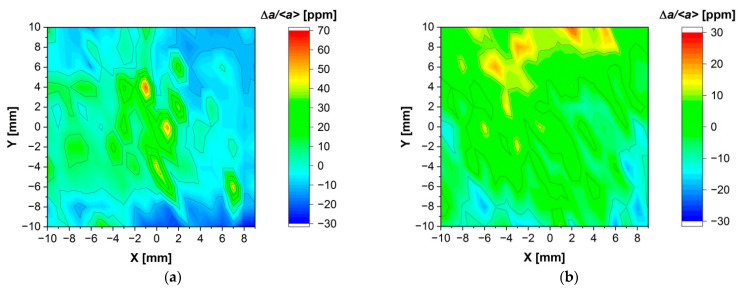
Lattice constant maps. (**a**) Cd_0.95_Mn_0.05_Te; (**b**) Cd_0.93_Mn_0.07_Te_0.98_Se_0.02_.

We conducted lattice constant mapping on monocrystalline samples of (Cd,Mn)Te (Figure 4a) and (Cd,Mn)(Te,Se) (Figure 4b). The lattice constant changes, denoted in Figure 4 as Δa/<a>, are expressed as in Equation (1):

(1) $$\frac{\Delta a}{\langle a\rangle }=\frac{a-\langle a\rangle }{\langle a\rangle }\cdot 10^{6}\;\left[ppm\right]$$

where a is the local value of the lattice constant and <a> is the arithmetic mean value of all local values of lattice constant, a, determined at different locations along the sample.

The average value of the lattice constant for Cd_0.95_Mn_0.05_Te is 6.47658 Å, and for Cd_0.93_Mn_0.07_Te_0.98_Se_0.02_ it is 6.46411 Å, both with a standard deviation of 0.00008 Å. In Figure 4, the deviations from the average lattice constant value along the sample are depicted as very small, in the order of parts per million (ppm). This indicates that both crystals exhibit a high level of uniformity in terms of lattice constant distribution. The results provide a very solid foundation for further research on these crystals.

#### 3.2.3. Presence of Blocks/Grains and Their Mutual Misorientation

**Figure 5:**
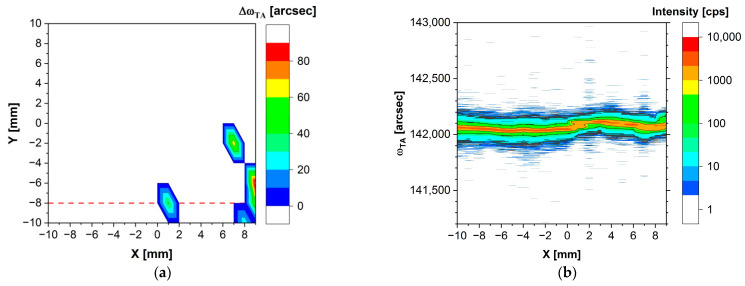
Cd_0.95_Mn_0.05_Te results. (**a**) Delta omega map of Cd_0.95_Mn_0.05_Te in the triple axis mode. (**b**) The map of the intensity of omega values, ω_TA_, obtained from 20 omega scans conducted along the Y = −8 line, with a 1 mm X step. The Y = −8 line is marked in Figure 5a with a red dashed line.

Delta omega maps were prepared to check the presence of blocks/grains in our samples and, if they were present, to determine their mutual misorientation. A delta omega map of the (Cd,Mn)Te sample is depicted in Figure 5a. A non-zero delta omega value indicates the presence of blocks (consisting of two or more) that exhibit misorientation relative to each other, with the delta omega value representing the maximum misorientation between these blocks. Conversely, a delta omega value of zero signifies that, at that specific measurement point, only a single peak was recorded, indicating the absence of blocks or grains.

In Figure 5a, a delta omega value of zero is found in the majority of measurement points. Out of the 220 points on the delta omega map, only 5 points show an omega scan curve with two peaks, indicating the presence of two misoriented blocks. This indicates that in 215 measurement points, which accounts for approximately 98% of the 18 × 20 mm^2^ area of this (Cd,Mn)Te sample, a single peak was recorded, implying the absence of grains or blocks with different orientations. Consequently, it can be concluded that a well-defined monocrystal is observed within the resolution limits of our measurement method.

The map in Figure 5b illustrates how the intensity (signal strength) of specific omega angles is varied in this (Cd,Mn)Te sample. This map was created using data from 20 omega curves collected at 20 points in the sample, along the Y = −8 line, with an X step of 1 mm. The aim of this was to visualize variations in the omega angle at different points (X, −8) within the sample. Here, the value of the omega angle should be determined based on the angle corresponding to the highest intensity, which signifies the peak of the signal. The choice of the Y = −8 line was motivated by the presence of several points with notably higher deviations, as clearly indicated in Figure 5a, where the Y = −8 line is marked by a red dashed line.

In Figure 5b, it is evident that the omega angle, depicted as points with the highest intensity, i.e., corresponding to the maximum of the omega curve, remains constant in the range from X = −10 to X = 0.5. At X = 0.5; a shift in the X-ray beam from one block (grain) to another is observed. Subsequently, from X = 2 to X = 9, the omega angle is once again maintained at a near-constant value. The maximum misorientation angle between these two blocks is measured at 50 arcsec. In the (Cd,Mn)Te sample, despite selecting a line along the Y-axis with poorer X-ray results for the creation of the map in Figure 5b, the omega angles remain nearly constant.

**Figure 6:**
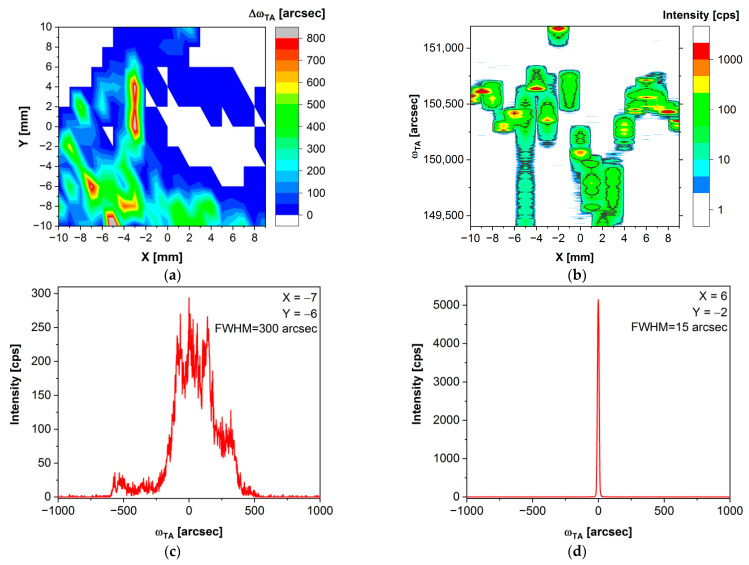
Cd_0.93_Mn_0.07_Te_0.98_Se_0.02_ results. (**a**) Delta omega map of Cd_0.93_Mn_0.07_Te_0.98_Se_0.02_ in the triple axis mode. (**b**) The map of the intensity of omega values, ω_TA_, obtained from 20 omega scans conducted along the Y = −10 line, with a 1 mm X step. (**c**) Omega scan for measurement point X, Y: (−7, −6). Four distinct maxima are visible, indicating a block-like or sub-grain structure of the sample. (**d**) Omega scan for measurement point X, Y: (6, −2). A narrow peak with an FWHM of 15 arcsec indicates a monocrystalline structure of the sample at that particular location. It is worth noting that the scale of the X axis for Figure 6c and d is identical. In the (Cd,Mn)(Te,Se) sample, there are areas with block-like structures (Figure 6c) as well as perfectly monocrystalline regions (Figure 6d).

Figure 6a depicts the delta omega map of a (Cd,Mn)(Te,Se) sample. This map was also measured in triple axis mode. In the case of the (Cd,Mn)(Te,Se) crystal, a higher number of measurement points with non-zero values of delta omega are observed, indicating the presence of multiple blocks or grains. Furthermore, the maximum misorientation between these blocks, represented by the delta omega value, is significantly larger compared to (Cd,Mn)Te, on the order of 100 arcsec (with a maximum of 800 arcsec), whereas for (Cd,Mn)Te, it was on the order of 10 arcsec (with a maximum of 90 arcsec).

The changes in the intensity of omega angle values in the omega scans of a (Cd,Mn)(Te,Se) sample conducted along the Y = −10 line are depicted in Figure 6b. In the case of the (Cd,Mn)(Te,Se) crystal, similar to (Cd,Mn)Te (Figure 5b), the map was generated based on 20 omega scans along the Y-axis, and a Y line was chosen where a greater number of measurement points with higher (worse) delta omega values were encountered. Here, a significant dispersion of omega angle values is evident. The variation in these values between the red areas from Figure 6b, those with the highest intensity, is 720–1080 arcsec (0.2–0.3 degrees). Let us recall that in the worst location of the (Cd,Mn)Te sample, Y = −8, variations in the omega angle were at the level of 50 arcsec (Figure 5b). Although the (Cd,Mn)(Te,Se) sample etched with Durose’s solution appeared to be monocrystalline to the naked eye, X-ray studies revealed the presence of misoriented blocks within it. This is clearly visible in Figure 6a, where monocrystalline regions, preferred for X and gamma radiation detectors, are highlighted in white and represent a small portion of the sample. The majority of the sample consists of blocks, which are less desirable in the aforementioned application because crystal structure defects serve as scattering or recombination centers for charge carriers.

Omega scans for selected points X, Y: (−7, −6) and (6, −2) are presented in Figure 6c and Figure 6d, respectively. Figure 6c is on the same angular scale as Figure 6d. In Figure 6c, four maxima can be observed, signifying a block-like or sub-grain structure of the sample. The FWHM value taken from the extreme maxima is 300 arcsec. Meanwhile, in Figure 6d, a single, very narrow peak with an FWHM of 15 arcsec is seen, indicating a monocrystalline structure of the sample at that particular location. In this paper, we observed the same issues that our (Cd,Mn)(Te,Se) crystals had previously encountered [38]. Although the sample appeared to be monocrystalline during visual observation of the Durose-etched surface, revealing no grains and twins, it is, in fact, composed of misoriented blocks with a significant mosaic component (areas with a surface on the order of square millimeters).

According to Darwin’s model [69], a monocrystal is composed of a mosaic (blocks) with sizes ranging from 10 nm to 1 µm, slightly misoriented with respect to each other. The angle of misorientation between blocks typically ranges from a few arcseconds to a few minutes, in exceptional cases a few degrees. These small-angle boundaries are formed by a set of dislocations.

A micro-mosaic is present in practically every crystal. However, delta omega maps of our (Cd,Mn)Te (Figure 5a) and (Cd,Mn)(Te,Se) (Figure 6a) samples indicate a significantly higher contribution of block-like structure in the second one. Furthermore, in the (Cd,Mn)(Te,Se) crystal, the maximum misorientation between the blocks, Δω_TA_, is 10 times larger than in the (Cd,Mn)Te crystal. Therefore, the results of omega scans suggest a more perfect crystalline structure in the crystal without selenium alloying, i.e., (Cd,Mn)Te.

#### 3.2.4. Full Width at Half Maximum (FWHM) of Omega Scans

**Figure 7:**
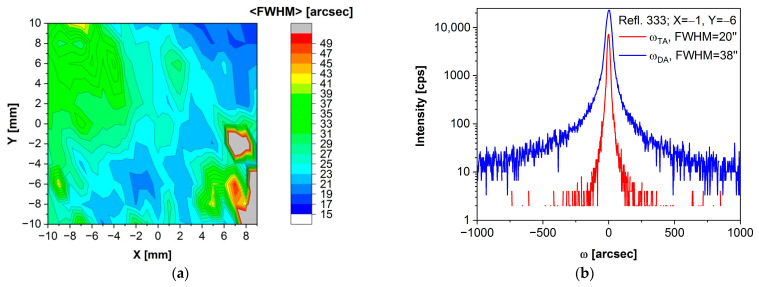
(**a**) Map of FWHM for Cd_0.95_Mn_0.05_Te acquired through omega scans conducted using triple-axis mode. (**b**) FWHM comparison at X = −1, Y = −6: 38 arcsec (double-axis, DA), 20 arcsec (triple-axis, TA).

Now, let us consider the (Cd,Mn)Te crystal once again. In Figure 7a, a map of the FWHM obtained from the omega scans at consecutive points of the sample is presented. This map was obtained in triple-axis mode. Several (five) points with worse (higher) FWHM values are located in the lower-right corner, which corresponds well to Figure 5a, where the presence of two blocks was recorded in that area. Apart from these exceptions, in the monocrystalline region of the sample, the FWHM of the omega scan is consistently better than ~50 arcsec.

In Figure 7b, a selected omega scan for the measurement point X = −1, Y = −6, chosen from the map in Figure 7a, has been presented. When measured in double-axis (DA) mode, meaning without the use of an analyzer, the FWHM of this rocking curve is 38 arcsec. However, when an analyzer is employed, i.e., in triple-axis mode, the FWHM is reduced to 20 arcsec.

An FWHM map is not presented for the (Cd,Mn)(Te,Se) crystal because the omega scans resulted in curves with multiple maxima (indicating the presence of blocks/grains in the sample). Therefore, it is challenging to arbitrarily determine which FWHM value should be included in the map.

For comparison, high-resolution rocking curve measurements of THM-grown (Cd,Zn)(Te,Se) crystals resulted in an FWHM value of 30.8 arcsec, and no mosaic structure was observed [70]. This outcome can be attributed to the THM method’s slower growth rate compared to the Bridgman method, mentioned in Section 1, which leads to fewer structural defects and, consequently, the achievement of a low FWHM value. On the other hand, when examining rocking curve studies of Bridgman-grown crystals, a broad spectrum of reported FWHM values for omega scans in the case of (Cd,Zn)Te exists, ranging from 8 to over 400 arcsec [71,72,73]. For as-grown (Cd,Mn)Te crystals, previous research has reported FWHM values of 68 arcsec [74] or 72 arcsec [75]. In contrast, our (Cd,Mn)Te crystal demonstrates superior performance, featuring an FWHM value for the omega scan that is almost two times smaller (in double-axis mode).

Our X-ray examinations suggest a better crystal structure in (Cd,Mn)Te crystals compared to (Cd,Mn)(Te,Se) crystals. The distribution of lattice constant in both samples was very good, exhibiting minimal changes at the ppm level. However, omega scans revealed a significant presence of block/grain-like structures in (Cd,Mn)(Te,Se) crystals, much higher than in (Cd,Mn)Te crystals, and displayed a higher degree of misorientation. Both X-ray studies and etch pit density measurements suggest that (Cd,Mn)Te crystals are more suitable for X-ray and gamma detectors compared to crystals with selenium addition.

#### 3.2.5. Tellurium Inclusions

**Figure 8:**
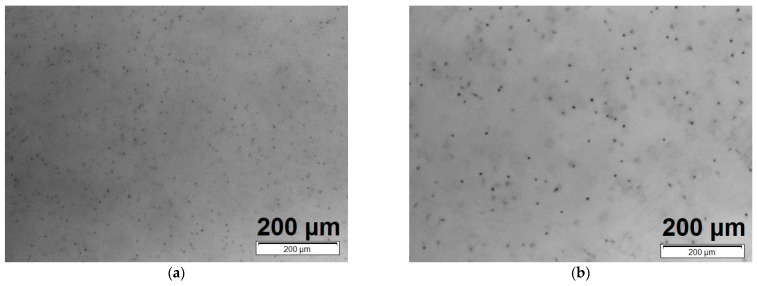
Infrared transmission images illustrating tellurium inclusions, observed as dark, spherical objects with sizes on the order of single micrometers. (**a**) Cd_0.95_Mn_0.05_Te. (**b**) Cd_0.95_Mn_0.05_Te_0.98_Se_0.02_.

Figure 8 shows tellurium inclusions observed through infrared transmission microscopy. These inclusions appear as dark, spherical objects with dimensions at the scale of single micrometers in both the crystals of (Cd,Mn)Te (Figure 8a) and (Cd,Mn)(Te,Se) (Figure 8b). The estimated density of tellurium inclusions is 6.35 × 10^5^ cm^−3^ in the (Cd,Mn)Te sample and 2.17 × 10^5^ cm^−3^ in the (Cd,Mn)(Te,Se) sample. In the crystal with selenium addition, an almost three times lower density of tellurium inclusions is observed. It is hypothesized that selenium plays a role in diminishing the protrusion of the retrograde solidus line near stoichiometry, thereby lowering the concentration of secondary phases rich in tellurium [7]. Our observations are consistent with the observations of (Cd,Zn)Te and (Cd,Zn)(Te,Se) crystals, where a decrease in the density of tellurium inclusions was also observed in crystals containing selenium compared to crystals without selenium [46].

### 3.3. Impact of Grain Boundaries and Twins

The influence of grain boundaries and twins on the FWHM of the omega curve measurement was investigated in (Cd,Mn)Te crystals. Specifically for this purpose, a selected (Cd,Mn)Te plate with both grain boundary and twin was examined. Studying grain boundaries and twins is crucial in CdTe-based materials for X-ray and gamma-ray detectors because understanding the structure of grain boundaries and twins can lead to improvements in the detector manufacturing process and the quality of X-ray and gamma-ray radiation detection, including the energy resolution of the detector.

**Figure 9:**
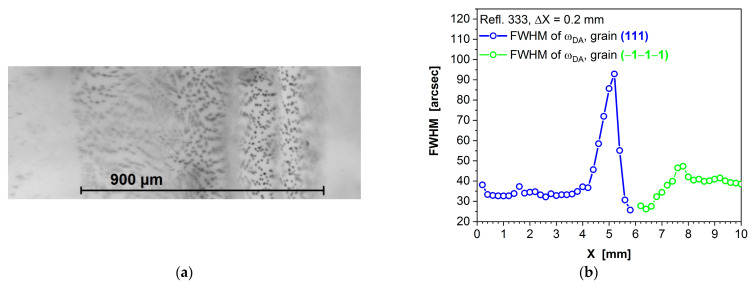
Harmful influence of grain boundary in (Cd,Mn)Te crystals. (**a**) Fused infrared images, focused at various depths of the sample, revealing the grain boundary width in this specimen. (**b**) FWHM of omega curve as a function of the measurement point along the sample. Due to the steep inclination of adjacent grains, the sample was scanned along a line from both sides of the grain boundary—from the left side of the grain boundary, corresponding to the blue points on the curve, as well as from the right side of the grain boundary, corresponding to the green points on the curve.

Figure 9a presents a compilation of several infrared (IR) images, each focused at different depths within the sample, in order to illustrate the width of the grain boundary. This grain boundary has a plane that is inclined relative to the imaging plane. The dark, spherical objects visible in Figure 9a correspond to tellurium inclusions situated within the grain boundary region, as investigated by us in references [76,77]. In this specific area, the width of the grain boundary measures 900 µm. Figure 9b shows changes in the FWHM values of the omega curves, recorded at intervals of 0.2 mm along the sample, as it transitions from one grain to another. Notably, the region where the FWHM undergoes a significant shift of approximately 60–70 arcsec spans a width of 1.6 mm. It is worth noting that the X-ray scan was conducted at a slightly different location compared to the IR image, which accounts for the variance in the grain boundary width values between the X-ray data and the IR image.

**Figure 10:**
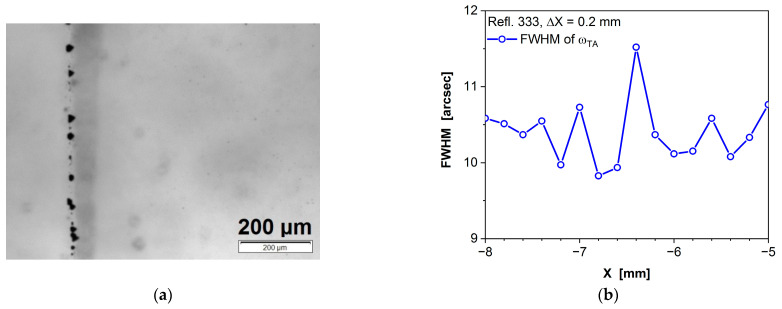
Negligible effect of twin on FWHM of omega curve in (Cd,Mn)Te crystal. (**a**) Infrared image of a twin decorated with tellurium inclusions, visible as dark objects aligning in a row on the left side of the image. (**b**) FWHM of omega curve as a function of the measurement point along the sample.

Figure 10a displays an IR image of a twin in (Cd,Mn)Te. This twin is decorated with tellurium inclusions, visible as dark objects arranged vertically on the left side of the image. The width of the twin measures approximately 70 µm and is one order of magnitude smaller than the width of the grain boundary shown in Figure 9a.

Figure 10b presents the FWHM values of the omega curves obtained during the scanning of a (111)-oriented sample with a 0.2 mm step, transitioning from one part of the twin to the other. In this case, the FWHM values are consistently below 12 arcsec, which is close to the limit of our diffractometer’s resolution in TA mode (8 arcsec). Importantly, no significant changes in FWHM values are observed within the standard error. This is because the two crystal parts separated by the twin exhibit high crystallographic quality in the measurement area, and they are rotated relative to each other around the normal to the (111) plane. As anticipated, the presence of the twin in the crystal does not seem to impact the FWHM, especially when the twin contains few defects.

Both techniques, infrared imaging and X-ray diffraction studies, underscore the detrimental impact of grain boundaries and the negligible effect of twins on the discussed properties of (Cd,Mn)Te crystals.

### 3.4. Photoluminescence Spectra of As-Grown and Annealed Crystals

**Figure 11:**
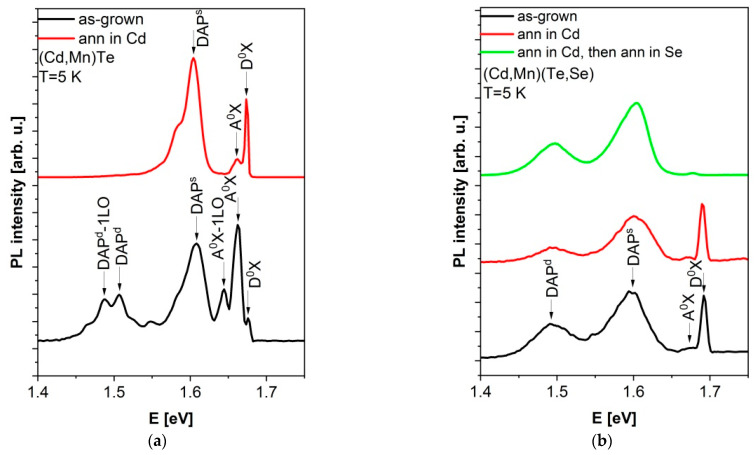
Photoluminescence spectra at a temperature of 5 K. (**a**) Cd_0.95_Mn_0.05_Te sample. Annealing in Cd vapors eliminated the DAP^d^ luminescence. (**b**) Cd_0.95_Mn_0.05_Te_0.98_Se_0.02_ sample. Annealing in Cd or Se vapors did not eliminate the DAP^s^ and DAP^d^ luminescence.

Figure 11a,b depict low-temperature photoluminescence (PL) spectra of (Cd,Mn)Te and (Cd,Mn)(Te,Se) samples, respectively. These spectra exhibit common features with the PL spectra of (Cd,Zn)Te. In both the (Cd,Mn)Te and (Cd,Mn)(Te,Se) samples we investigated, we can identify excitonic luminescence, donor–acceptor transitions, and defect-related bands, similar to what is observed in (Cd,Zn)Te [40].

In both of the as-grown materials, there are excitonic transitions, including D^0^X (exciton bound to a neutral donor) and A^0^X (exciton bound to a neutral acceptor), as well as two donor–acceptor pair transitions (DAP). In some instances, these transitions are accompanied by their phonon replicas, with energies approximately 20 meV lower. Specifically, shallow (s) and deep (d) DAP transitions are located about 70 meV and 200 meV below the exciton lines, respectively.

Bridgman-grown (Cd,Mn)Te and (Cd,Mn)(Te,Se) crystals naturally exhibit a high concentration of Cd vacancies, which act as acceptors. This is a consequence of the insufficient Cd content at high temperature during crystal growth (~1100 °C), caused by the high partial pressure of Cd. To reduce the concentration of cadmium vacancies, we applied annealing to both crystals, with and without selenium, in a cadmium-rich environment at 800 °C.

Consequently, in (Cd,Mn)Te crystals, the intensities of the A^0^X and DAP^s^ PL lines were reduced, and emission from a DAP^d^ transition was eliminated, as demonstrated in Figure 11a. Thus, it can be inferred that the concentration of acceptors is lower in the Cd-annealed sample compared to the as-grown one. Previous studies have shown that annealing in Cd vapors at 733 °C or 786 °C also effectively eliminates the PL peak associated with the DAP^s^ transition in our (Cd,Mn)Te samples (Figure 9 and Figure 10 in [78]).

Annealing of (Cd,Mn)(Te,Se) crystals in cadmium vapors also led to a reduction in the PL intensities of DAP^s^ and DAP^d^ lines, consequently decreasing the concentration of cadmium vacancy acceptors, which is presented in Figure 11b. However, this effect is notably less pronounced compared to (Cd,Mn)Te crystals. On the other hand, our prior research demonstrated that subjecting (Cd,Mn)(Te,Se) samples to double annealing in cadmium vapors had a negligible impact on the intensity of the DAP^s^ and DAP^d^ PL lines (as shown in Figure 9 in [38]).

Conversely, in the (Cd,Mn)(Te,Se) crystal, the annealing process in selenium vapors at 350 °C primarily affected the concentration of donors (Figure 11b). Evaluating the changes in PL intensities of DAP^s^ and DAP^d^ lines after annealing in selenium vapors presents a challenge due to the disappearance of the reference line, D^0^X. It is likely that the number of selenium vacancies, which should, in principle, act as deep donors (similar to tellurium vacancies [38,79,80]), has been reduced. Furthermore, in (Cd,Mn)(Te,Se), the DAP^d^ PL line is observed at higher temperatures, extending up to 120 K, compared to (Cd,Mn)Te, where it is observed up to 100 K. In both crystals, the DAP^s^ emission disappears at 60 K.

When comparing the PL spectra of both (Cd,Mn)Te and (Cd,Mn)(Te,Se) crystals, it can be observed that in (Cd,Mn)Te crystals, changes in the intensities of PL lines associated with acceptors (A^0^X, DAP) and, consequently, changes in the concentration of acceptors (cadmium vacancies) after annealing in cadmium vapors are more noticeable than in Cd-annealed (Cd,Mn)(Te,Se) crystals. In (Cd,Mn)(Te,Se) crystals, there may exist complexes containing selenium vacancies, which create deep energy levels, i.e., deep traps for charge carriers. This could potentially explain the difficulties encountered in eliminating the DAP^s^ and DAP^d^ PL lines in the spectra of crystals containing selenium.

### 3.5. Detector Response

**Figure 12:**
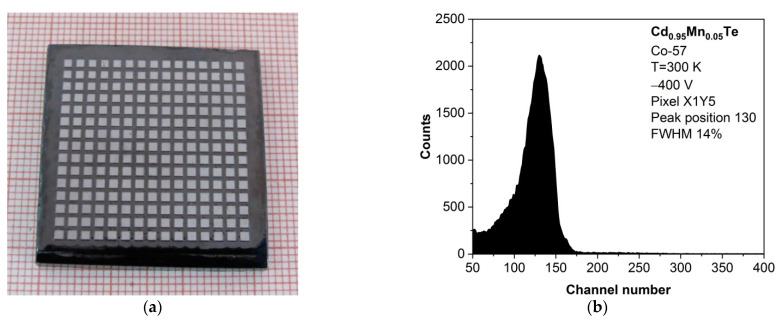
As-grown Cd_0.95_Mn_0.05_Te pixelated detector. (**a**) A photograph of the detector with dimensions of 32 × 32 × 4.3 mm^3^ and 225 pixels. (**b**) Spectroscopic performance from a selected pixel made at room temperature using a Co-57 source. The cathode was biased with −400 V. The peak in the spectrum is related to 122 keV.

Finally, a comparison was made between the detector responses of two materials, (Cd,Mn)Te and (Cd,Mn)(Te,Se). The performance of the detectors at room temperature was assessed using a Co-57 point source. An example image of a (Cd,Mn)Te pixelated detector, which was prepared in our laboratory, is shown in Figure 12a.

Our as-grown (Cd,Mn)Te detector can detect 122 keV gamma-rays emitted by Co-57, demonstrating an as-measured energy resolution that ranges from 8% to 17%, depending on the pixel. The spectroscopic performance of a selected (Cd,Mn)Te detector pixel, featuring an FWHM of 14%, is presented in Figure 12b. Despite the high degree of homogeneity exhibited by (Cd,Mn)Te crystals, especially in X-ray measurements, the energy resolution of individual pixels within the same detector can vary up to two-fold. In our measurements, we did not employ any calibration techniques to correct for pixel-to-pixel variations in energy response. In the future, it would be worth considering implementing such enhancements in our system. Our detectors also lacked guard rings surrounding the pixels to minimize side leakage current.

Conversely, our (Cd,Mn)(Te,Se) detector only detects X-rays from Co-57 at 7 keV with an energy resolution of approximately 45%, along with a minor trace of gamma-rays at 14.4 keV. The reduced performance of our (Cd,Mn)(Te,Se) detector may be associated with the presence of a deep trap contributing to the luminescence of DAP^d^ and a substantial presence of blocks in the crystal structure, although further investigation is needed to confirm this hypothesis.

Let us compare the energy resolutions of some detectors described in the literature and constructed using (Cd,Mn)Te crystals obtained through the Bridgman method. The energy resolution of a planar as-grown Cd_0.9_Mn_0.1_Te detector with a guard ring, biased at 360 V, and characterized by an electron μτ product on the order of 10^−4^ cm^2^V^−1^ is in the range of 25–30% for 59.5 keV gamma rays from an Am-241 radiation source [24]. Another example is a planar Cd_0.95_Mn_0.05_Te detector biased at 150 V, which has an energy resolution of 9.2% FWHM for the 59.5 keV gamma peak of Am-241. This sample had an electron μτ product of 1.7 × 10^−3^ cm^2^V^−1^ [19]. Finally, a Frisch grid Cd_0.95_Mn_0.05_Te detector biased at 2900 V achieves an energy resolution of 7.5% FWHM at 662 keV of Cs-137. The μτ product value for electrons was 1.7 × 10^−3^ cm^2^V^−1^ in this sample [81]. The resolution of our (Cd,Mn)Te detector aligns with the trends reported in the literature.

In the case of a planar detector constructed with a crystal of Cd_0.95_Mn_0.05_Te_0.98_Se_0.02_, obtained using the vertical Bridgman method and characterized by an electron μτ product equal to 1.29 × 10^−3^ cm^2^V^−1^, its energy resolution was measured at 11% for 59.5 keV gamma rays from an Am-241 source when biased at 240 V [25].

Concerning detectors constructed with (Cd,Zn)Te, their technology is significantly more advanced than detectors built with (Cd,Mn)Te. The latest research reports on the energy resolution capabilities in a linear array pixel detector based on (Cd,Zn)Te grown by the boron oxide encapsulated vertical Bridgman method (B-VB), achieving an FWHM of 0.96% at 122 keV from Co-57 when biased at 700 V [82]. However, in that study, researchers are paying significant attention to dedicated electronics that could utilize the full potential of (Cd,Zn)Te crystals, i.e., improving their performance. In another instance, a (Cd,Zn)Te pixel detector, also grown by the B-VB method, exhibited an energy resolution with an FWHM of 1.3% at 122 keV when biased at 900 V. The estimated μτ product for electrons in this detector was in the range of 0.6–0.7 × 10^−3^ cm^2^V^−1^ [83].

If we consider detectors built with (Cd,Zn)(Te,Se) crystals, most of the data are related to crystals obtained through the THM method. For instance, a detector in a Frisch grid configuration with a composition of 10% Zn and 2% Se, where the electron μτ parameter was 6.6 × 10^−3^ cm^2^V^−1^, exhibited a resolution of 1% FWHM at 662 keV when biased at 3000 V [7,46].

Let us emphasize the motivation for working on a relatively new material in X- and gamma-ray detector applications, (Cd,Mn)Te, instead of (Cd,Zn)Te. Despite significant improvements made in the technology of detectors built on (Cd,Zn)Te over the past thirty years, this technology still has limitations due to the presence of defects such as tellurium inclusions and sub-grain boundaries, which negatively impact the performance of these detectors [63]. Furthermore, the segregation coefficient of Zn in CdTe is 1.3 [51], causing the first-to-freeze part of the crystal to have a different chemical composition than the last-to-freeze part. Typically, plates from the central region of such a crystal are chosen, while the edge parts are discarded. Therefore, researchers have directed their focus towards exploring new materials, including the introduction of Se into (Cd,Zn)Te, a modification that appears to address the abovementioned detrimental defects in (Cd,Zn)Te. Moreover, a notable advantage of the (Cd,Mn)Te, over (Cd,Zn)Te, lies in its ability to obtain large single crystals, with dimensions of approximately 30 × 30 × 3–5 mm^3^, as reported in this work. It is essential to employ large detector volumes, ensuring that a maximum number of incident photons have the chance to interact within the detector to achieve high sensitivity and efficiency [40]. Finally, in (Cd,Mn)Te crystals, segregation is negligibly small [50]. As a result, from a single ingot, we could obtain few crystal plates for detectors, potentially leading to reduced production costs in the future.

In this study, we compare materials obtained using the Bridgman method. Our multiple crystallization processes and measurements of crystal properties clearly indicate that larger monoblocks grow in (Cd,Mn)Te and (Cd,Mn)(Te,Se) crystals compared to (Cd,Zn)Te, and that (Cd,Mn)Te detectors are capable of detecting X-ray and gamma radiation from a Co-57 source, while our (Cd,Mn)(Te,Se) crystals exhibit greater challenges in this regard. Of course, there is room for improvement in (Cd,Mn)Te and (Cd,Mn)(Te,Se) crystals. We suspect that the currently non-competitive energy resolution of our (Cd,Mn)Te detector compared to (Cd,Zn)Te detectors demonstrated by other researchers may stem from the insufficient purity of our crystals, despite their very good crystal structure. Perhaps, even though we purify manganese to a purity of 6N, it contains a substantial amount of oxygen that is challenging to remove. In the near future, we plan to enhance the purity of our crystals by applying the THM method instead of the Bridgman method.

## 4. Conclusions

We conducted a comparative analysis of two CdTe-based compounds, (Cd,Mn)Te and (Cd,Mn)(Te,Se), both grown using the Bridgman method, focusing on their crystal structure, hardness, luminescence properties, and effectiveness as X-ray and gamma-ray detectors.

X-ray examinations of visually identified monocrystalline samples revealed very uniform lattice constants in both crystals, with minimal variations at the ppm level. However, omega curve measurements unveiled a significant presence of block-like structures within (Cd,Mn)(Te,Se) crystals, resulting in delta omega values, corresponding to the maximum misorientation between blocks, on the order of 100 arcsec (with a peak at 800 arcsec). In contrast, (Cd,Mn)Te crystals exhibited nearly perfect monocrystalline structures, with block-like features observed in only 2% of the 18 × 20 mm^2^ area. Additionally, the misorientation angles between blocks in (Cd,Mn)Te were approximately ten times smaller than those observed in the selenium-containing crystals. Etching the crystals with Inoue solution further emphasized this contrast, displaying one order of magnitude fewer etch pits in (Cd,Mn)Te compared to (Cd,Mn)(Te,Se). The study also highlighted the detrimental influence of grain boundaries and the negligible impact of twins on the crystal structure quality of our samples.

We find that (Cd,Mn)Te shows greater promise as a material for X-ray and gamma-ray detectors when compared to our (Cd,Mn)(Te,Se). The (Cd,Mn)Te detector can detect 122 keV gamma rays from a Co-57 source, achieving an energy resolution ranging from 8% to 17%, depending on the pixel. Conversely, our (Cd,Mn)(Te,Se) detector exhibited poor responses to X- and gamma-rays, potentially due to the presence of a deep trap involved in DAP^d^ luminescence, which cannot be eliminated through annealing in Cd vapors, unlike in the case of (Cd,Mn)Te. Additionally, the significant contribution of block-like structures in selenium-containing crystal samples, accompanied by notably larger misorientation angles between these blocks compared to (Cd,Mn)Te, may contribute to the bad performance.

## Data Availability

The data presented in this study are available on request from the corresponding author.
